# Ecology and management of grapevine leafroll disease

**DOI:** 10.3389/fmicb.2013.00094

**Published:** 2013-04-24

**Authors:** Rodrigo P. P. Almeida, Kent M. Daane, Vaughn A. Bell, G. Kai Blaisdell, Monica L. Cooper, Etienne Herrbach, Gerhard Pietersen

**Affiliations:** ^1^Department of Environmental Science, Policy and Management, University of California at BerkeleyBerkeley, CA, USA; ^2^The New Zealand Institute for Plant and Food Research LimitedHavelock North, New Zealand; ^3^Division of Agriculture and Natural Resources, University of California at NapaNapa, CA, USA; ^4^UMR1131 Santé de la Vigne et Qualité du Vin, Institut National de la Recherche AgronomiqueColmar, France; ^5^UMR1131, Université de StrasbourgStrasbourg, France; ^6^Agricultural Research Council-Plant Protection Research Institute, c/o Department of Microbiology and Plant Pathology, University of Pretoria, Pretoria, South Africa

**Keywords:** grapevine disease, Closteroviridae, vector, mealybug, integrated pest management

## Abstract

Grapevine leafroll disease (GLD) is caused by a complex of vector-borne virus species in the family Closteroviridae. GLD is present in all grape-growing regions of the world, primarily affecting wine grape varieties. The disease has emerged in the last two decades as one of the major factors affecting grape fruit quality, leading to research efforts aimed at reducing its economic impact. Most research has focused on the pathogens themselves, such as improved detection protocols, with limited work directed toward disease ecology and the development of management practices. Here we discuss the ecology and management of GLD, focusing primarily on *Grapevine leafroll-associated virus 3*, the most important virus species within the complex. We contextualize research done on this system within an ecological framework that forms the backbone of the discussion regarding current and potential GLD management strategies. To reach this goal, we introduce various aspects of GLD biology and ecology, followed by disease management case studies from four different countries and continents (South Africa, New Zealand, California-USA, and France). We review ongoing regional efforts that serve as models for improved strategies to control this economically important and worldwide disease, highlighting scientific gaps that must be filled for the development of knowledge-based sustainable GLD management practices.

## INTRODUCTION

Emerging plant diseases are a global threat to the food supply, environmental sustainability, and economic stability of regions and nations. In this paper, we discuss the ecology and management of grapevine leafroll disease (GLD), a worldwide disease that is caused by a complex of virus species in the family Closteroviridae, which contains emerging and re-emerging plant pathogens of economic importance. GLD is present in virtually all commercial grape (*Vitis vinifera*) growing regions; its distribution is thought to be due to regional, continental, and intercontinental transport of virus-infected plant material. While GLD has long been present in the major grape-growing regions, it has only recently been recognized as a disease of economic importance. Various hypotheses have been proposed to explain this (e.g.,Golino et al., 2008), but none have been well supported. For example, there is no evidence of the emergence of a new virus species or strain (Wang et al., 2011), or introduction of a rapidly moving or efficient insect vector species associated with the increased incidence of GLD. The only common factors are the observation of vector-mediated pathogen spread in vineyards and an increased GLD awareness by academics, farmers, and other stakeholders. Regardless of the driving forces, GLD is now considered a disease of importance in viticulture, especially to wine grape growers who aim for a high quality uniform crop.

Herein, we will not focus on factors that have made GLD such a pre-eminent disease, although studies are needed to address this. We propose that the integration of disciplines is necessary to address GLD, and to devise disease management practices that are practical, sustainable, financially viable, and environmentally sound. Within this interdisciplinary context, our goal is to discuss various components of GLD that are relevant to its ecology, epidemiology, and management. Much of this review focuses on the mealybug-transmitted ampeloviruses, more specifically *Grapevine leafroll-associated virus 3* (GLRaV-3), which is the most widespread species in the virus complex causing GLD. We highlight notable gaps in the current body of knowledge that need to be addressed for the development of sustainable disease control practices. Then we discuss management strategies being implemented in each of four countries in four continents by presenting case studies.

## GRAPEVINE LEAFROLL DISEASE

Grapevine leafroll disease has been described from different regions in Europe and elsewhere for over a century (Hoefert and Gifford, 1967), and was first shown to be transmissible to vines in 1936 (Scheu, 1936). The demonstration of graft transmissibility opened early avenues of GLD research, including the search for etiological agents and the impact of abiotic factors on symptom development. Even today the etiology and symptomatology of GLD is not completely clear, as multiple virus species cause GLD, and symptoms result from complex biotic and abiotic interactions. Furthermore, there is no infectious clone for any agent associated with GLD.

**FIGURE 1:**
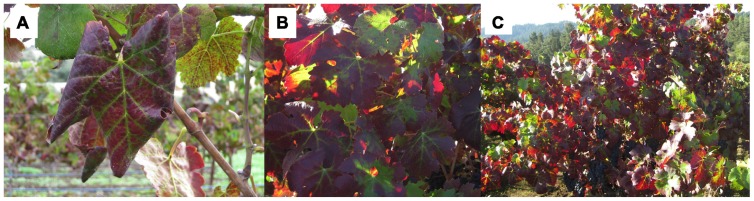
**Leaf symptoms of grapevine leafroll disease include inter-veinal reddening and leafrolling in red-fruited varieties.** Symptoms are most pronounced around the harvest period. These photographs were taken in the fall (September) in Napa, CA, USA. Photographs show symptomatic leaf **(A)**, group of leaves **(B)**, and whole plant **(C)**.

Grapevine leafroll disease is most obvious and problematic in cool-climate regions, where fruit on infected vines has delayed ripening that results in lowered brix, which in turn affects wine quality (Over de Linden and Chamberlain, 1970;Goheen, 1988). The most obvious GLD symptoms appear in the fall, when red cultivars display leaf reddening with green venation (**Figure 1**). While symptoms are not as apparent in white cultivars, there is a slight leaf chlorosis. Both red and white cultivars develop downward rolling of leaf margins and phloem disruption. Significant losses result from a combination of factors including yield reductions of up to 40%, increased management costs, shortened vineyard life spans, and adverse impacts on wine quality resulting from decreased fruit quality and delayed maturation (Woodrum et al., 1984;Goheen, 1988;Credi and Babini, 1997;Martelli et al., 2002). The economic impact of GLD is still poorly understood, as are the implications of various control strategies. A recent study byAtallah et al. (2012) estimated the economic impact of GLD to range from US$25,000 to US$40,000 per hectare for vineyards with a 25-year lifespan. The authors analyzed various scenarios, incorporating disease prevalence, yield reduction and fruit quality; at low levels of disease incidence (1–25%), roguing can significantly decrease economic losses, which was identified as an economically important practice together with planting of virus-free plant material. The economic impact of vector management has not been explored.

Grapevine leafroll disease has three essential biological components: (1) a complex of viruses in the Closteroviridae, (2) grapevine host plants, and (3) species of mealybugs (Pseudococcidae) and soft scales (Coccidae) that transmit GLRaVs. Much of this review will focus on GLRaV-3, which is the best studied species worldwide and has been implicated in a majority of GLD spread that has been mediated by known insect vectors. While GLRaV-2 is of economic importance, this *Closterovirus* species has no known vectors (Martelli et al., 2002). In addition, GLRaV-7, a member of the proposed genus Velarivirus (Al Rwahnih et al., 2012), does not appear to cause GLD and also has no known vectors (Tsai et al., 2010).

## GRAPEVINE LEAFROLL-ASSOCIATED VIRUSES

Virus species causing GLD are sequentially named *Grapevine leafroll-associated virus 1*, *Grapevine leafroll-associated virus 2*, and so on (GLRaV-1, GLRaV-2, GLRaV-n). All GLRaVs are in the genus *Ampelovirus*, except for GLRaV-2 and GLRaV-7, as previously discussed. GLRaVs in the genus *Ampelovirus* are divided into two phylogenetic groups, one that includes GLRaV-4, -5, -6, -9, and others, and another comprising GLRaV-1 and -3 (Maliogka et al., 2009). The taxonomy of GLRaVs is undergoing significant changes with recent proposals awaiting International Committee on Taxonomy of Viruses (ICTV) approval; the most relevant proposal is a change in sequence similarity thresholds for delineating species that would collapse GLRaV-4, -5, -6, -9, and other proposed species and divergent variants into one species, GLRaV-4 (Martelli et al., 2012;Thompson et al., 2012).

Both groups of GLRaV ampeloviruses, like other species in the Closteroviridae, are filamentous virions with a large (13–18 kb) positive-sense single-stranded RNA genome (Fuchs et al., 2009;Martelli et al., 2012). However, there are important differences in genome structure between the groups. The genomes of GLRaV-4-like species are ~5 kb smaller and lack several open reading frames on their 3^′^ ends that are present in GLRaV-1 and -3 (Thompson et al., 2012). Despite the large genetic diversity among GLRaV species, little is known about the phenotypic variability in disease symptoms among or within species. One careful study of GLRaV-2 demonstrated that disease symptoms were associated with the phylogenetic clustering of variants (Bertazzon et al., 2010), but similar work has not been performed with other viruses. Despite this gap in knowledge, GLRaV-3 has emerged as the key species causing GLD worldwide. The reasons behind the prominence of GLRaV-3 are poorly understood, especially because other GLD-causing species also co-exist with GLRaV-3, often within one vineyard or plant (Sharma et al., 2011), and some can be transmitted by the same vector species (Le Maguet et al., 2012). Notably, GLRaV-3 has been identified as the main species being transmitted by vectors throughout the world (see case studies).

The importance of GLRaV-3 genetic diversity is not understood from a phenotypic or ecological perspective. However, some important insights into GLRaV-3 ecology have been gained from genetic diversity studies. First, it appears that most variants are present in major grape-growing regions worldwide (Gouveia et al., 2011;Jooste et al., 2011;Sharma et al., 2011). Second, it is likely that much of the diversity within the species has yet to be discovered, given the increasing number of well-supported phylogenetic clades (e.g.,Sharma et al., 2011;Seah et al., 2012). Lastly, there is no evidence of positive selection in GLRaV-3 field populations (Wang et al., 2011), suggesting that the virus is not undergoing novel selective pressures.

## HOST PLANTS

Although ampeloviruses colonize a wide range of plant taxa, GLRaVs appear to be limited to grapevines (*Vitis*). To our knowledge, GLRaVs have only been isolated from *Vitis* spp. Focus on the commercially widespread *Vitis* *vinifera* may have limited our knowledge of potential host range, although a recent survey in Napa Valley, California, which included 41 plant species in 12 families in addition to wild grapes (*Vitis californica* and *Vitis californica* × *Vitis vinifera* hybrids), showed that wild *Vitis* can be infected with GLRaV-2 and GLRaV-3 (Klaassen et al., 2011). Because of extensive exchange of easily propagated plant material that has occurred worldwide (Rowhani et al., 2005), transport of virus-infected plant material has been identified as a major factor responsible for the global spread of GLD and its etiological agents. Quarantine regulations and national programs aimed at reducing the import of pathogens have been established in several countries, and are responsible for providing virus-free plant material to farmers. The integration of these practices into management of GLD is discussed below.

## INSECT VECTORS

Plant to plant transmission of GLRaV-3 by the mealybug *Planococcus ficus* (Signoret) was the first demonstration of an insect vector of a GLD pathogen (Engelbrecht and Kasdorf, 1990). Since then, several species of mealybugs have been shown to transmit GLRaV species, including *Pseudococcus maritimus* (Ehrhorn), *Pseudococcus viburni* (Signoret), *Pseudococcus longispinus* (Targioni-Tozzetti), *Pseudococcus calceolariae* (Maskell), *Pseudococcus comstocki* (Kuwana), *Planococcus citri* (Risso), *Phenacoccus aceris* (Signoret), and *Heliococcus bohemicus* Sulc (reviewed inDaane et al., 2012;Herrbach et al., 2013). Additionally, the soft scales *Pulvinaria vitis* (L.), *Parthenolecanium corni* (Bouché), *Ceroplastes rusci* (L.), *Neopulvinaria innumerabilis* (Rathvon), *Coccus longulus* (Douglas), *Parasaissetia nigra* (Nietner), and *Saissetia* sp. are also vectors (Belli et al., 1994;Mahfoudhi et al., 2009;Le Maguet, 2012;Herrbach et al., 2013;Krüger and Douglas, 2013). Impressive here is the breadth of vector species, which is essentially inclusive of all common mealybugs and soft scales found worldwide where GLD is of concern.

Recognition of insect vectors is essential for the development of disease management practices, including control of the correct vector species. However, the ecological relevance of different mealybug or soft scale species to GLD spread has yet to be properly addressed.Tsai et al. (2010) found no evidence of strict vector–virus species specificity for transmission and, to date, it appears that all GLRaV species can be transmitted by the different grape-associated mealybug species tested. This hypothesis was further supported with the demonstration that *Ph. aceris* transmits six *Ampelovirus* species (Le Maguet et al., 2012). Therefore, all mealybugs colonizing grapevines should be considered potential GLRaV vectors until proven otherwise, and vector biology rather than species becomes most important.

Vineyard mealybugs generally have four larval instars for the female and five for the male (Ben-Dov, 1995). The small (~0.5 mm), unsettled first instar, or crawler, is considered to be the dispersal stage, and can be easily moved on personnel, equipment, infested nursery stock (Daane et al., 2012), and carried by the wind (Barrass et al., 1994). Whereas all mealybug and soft scale life stages may be capable of transmitting GLRaV-3, the younger nymphs appear to be more efficient (Petersen and Charles, 1997;Tsai et al., 2008). Vector species with more annual generations or higher fecundity would pose a greater threat. Variability in annual number of generations and fecundity exists. For example, in coastal California wine grape vineyards there are approximately one, two, three, and four annual generations of *Pa. corni*, *Ps. maritimus*, *Ps. viburni*, and *Pl. ficus*, respectively (Geiger and Daane, 2001;Gutierrez et al., 2008;Varela et al., 2013).

Mealybugs and soft scales are phloem feeders that use long, slender mouthparts to suck plant fluids (Daane et al., 2012). Most vineyard mealybug species can feed on the trunk, canes, leaves, and berries; however, there is variation in seasonal feeding location and movement on the vine among and within species, as described for *Ps. maritimus* (Geiger and Daane, 2001;Grasswitz and James, 2008), *Pl. citri* (Cid et al., 2010), and *Pl. ficus* (Becerra et al., 2006). Some mealybug species commonly maintain a portion of their population on vine roots, such as *Ps. calceolariae* (Bell et al., 2009) and *Pl. ficus* (Walton and Pringle, 2004). This presents a considerable replant problem as after the vine is removed, remnant roots can remain viable for years, supporting GLRaVs and mealybugs that bridge the old infested vineyard to the new replants (Pietersen, 2006).

Control of different vector species can vary considerably. Monitoring insect populations is an essential component of pest control; however, visually monitoring for mealybugs, especially at low densities, is too labor intensive to be cost effective. Sex pheromones for numerous species have recently been identified, including *Pl. ficus*, *Ps. viburni*, *Ps. maritimus*, *Ps. longispinus*, and *Ps. calceolariae* (reviewed inDaane et al., 2012), and trap counts can be used to predict berry damage (Walton and Pringle, 2004); however, there are no economic injury levels determined for these insects as GLD vectors. To control GLD spread, most vineyard managers have adopted a zero tolerance for vectors, and monitoring manifests as presence/absence scores. Efficient insecticides for mealybugs and soft scales exist, particularly some neonicotinoids and biosynthesis inhibitors (Daane et al., 2012). However, *Pl. ficus* first instar nymphs can both acquire and inoculate GLRaV-3 in less than 1 h (Tsai et al., 2008). Because the more effective insecticides are systemic, and the vector must feed on the plant to be killed, the applications may reduce mealybug densities in the treated vineyard but not necessarily protect it from virus spread by dispersing mealybugs. For some mealybug species, insecticides alone do not provide complete control, and additional control is provided by natural enemies. In New Zealand, for example, *Ps. viburni* was brought under exceptional control by release of the parasitoid *Acerophagus* (*Pseudaphycus*)* maculipennis* Signoret (Charles et al., 2010). In contrast, *Anagyrus pseudococci* Signoret is the primary parasitoid of *Pl. citri* and *Pl. ficus* around the world (Daane et al., 2012), but parasitism alone does not deliver control sufficient to reduce the spread of GLD. Mating disruption, which works best at lower pest densities, is being investigated for *Pl. ficus* (Walton et al., 2006) and may become an integral part of future control measures.

Of those countries reported in this review, *Pl. ficus* and *Ps. calceolariae* appear to be of greatest concern in most regions, but all mealybugs and soft scales should be viewed as potential vectors. The role played by different vector species in GLD epidemiology and vector ecology is still poorly understood. Regardless of the species, for GLD management through vector control, it is likely that multiple monitoring and control techniques must be employed to maintain the exceptionally low pest densities needed to suppress and control GLD.

## VIRUS TRANSMISSION BIOLOGY

Among insect-borne plant viruses, those transmitted by mealybugs and soft scales are among the least understood. These insects transmit other viruses of economic importance to a range of crops such as cassava, banana, pineapple, and cocoa (Herrbach et al., 2013). The characterization of transmission parameters has rarely been performed, severely limiting our understanding of disease epidemiology. Nevertheless, the importance in understanding the transmission of ampeloviruses infecting grapevines has recently become apparent, and several insect vectors species are now being studied for their efficiency to transmit different GLRaV species. Most work did not go beyond the identification of new insect species as vectors. In many cases virus source plants were infected with multiple virus species, which presents a challenge because multiple infections may lead to cases of virus facilitation or competition. While a better picture of GLRaV transmission by vectors is emerging, much remains undone. Nevertheless, trends can be inferred and used to generate testable hypotheses. The transmission of GLRaV-3 appears to be more efficient than that of other GLRaV species; based on inferences from studies designed to identify new vector–virus combinations rather than compare transmission efficiency. Competing hypotheses may explain these observations. First, viruses that are transmitted less efficiently may reach lower populations within plants than GLRaV-3. Therefore they may be acquired less frequently from the phloem, resulting in lower transmission rates. Alternatively, molecular interactions between virus and vector may affect transmission efficiency. Lastly, GLRaVs may be transmitted with similar efficiency, but those with observed lower transmission may require a higher number of virions inoculated to generate a successful infection.

In the Closteroviridae, all vector-borne viruses studied so far are transmitted in a semi-persistent manner (Karasev, 2000), but in this regard GLRaVs are poorly characterized.Cabaleiro and Segura (1997b) provide important insights into the biology of GLRaV-3 transmission by *Pl. citri*; however, they mentioned that their results were not conclusive to characterize transmission as semi-persistent. Conclusive evidence of semi-persistent transmission of GLRaV-3 was only obtained byTsai et al. (2008). Transmission efficiency of GLRaV-3 by *Pl. ficus* first instars peaked with 24-h acquisition and inoculation access periods, with a leveling-off after 48 h (Tsai et al., 2008). *Pl. ficus* mealybugs lost the ability to transmit GLRaV-3 four days after acquisition (Tsai et al., 2008). It is imperative that similar experiments with more virus and vector species be performed, although given the phylogenetics of the group (Tsai et al., 2010), it is expected that all GLRaV ampeloviruses will be transmitted in a semi-persistent manner.

**FIGURE 2:**
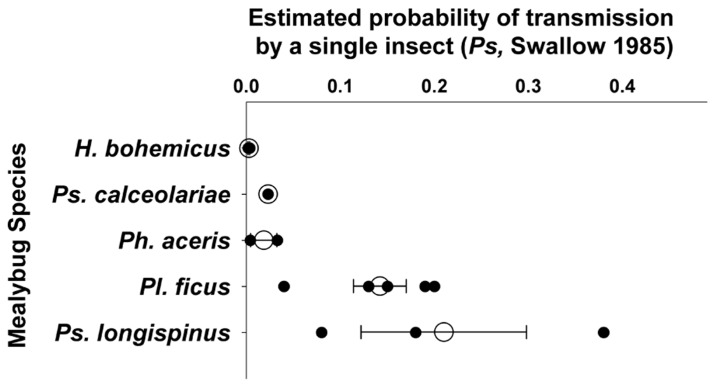
**Estimated transmission efficiency of GLRaV-3 per individual per day (*P*s; perSwallow, 1985) by different mealybug species, including studies that tested transmission by first and/or second instars, nymphs, adults, or mixed life stages, and used access periods of 1 day or longer**. When available, only earliest life stages tested are included. From individual publications including multiple experiments, those experiments are combined to produce one estimate of *P*s for each mealybug species. Results are based on a limited number of peer-reviewed publications per species; *Pl. ficus* – 5, *Ps. longispinus* – 3, *H. bohemicus* and *Ph. aceris* – 2, and *Ps. calceolariae* – 1. Figure shows *P*s for individual publications (dark circles), mean (open circles), and standard error.

Reported transmission rates are difficult to compare given the varied experimental methods and generally low number of replicates used. For example, with semi-persistent transmission, a vector can lose the ability to transmit a virus upon molting to the next life stage, and longer experimental acquisition access periods used may have resulted in insects being moved to a new plant immediately after molting (and losing acquired virus). Such a protocol would effectively result in a shorter acquisition access period. Here, we report calculated *P*s values, followingSwallow (1985), which provide an estimate of infection rate or probability of transmission by a single insect derived from experiments that used insect groups (**Figure 2**), based on existing transmission studies. When any one particular published study included multiple experiments, we combined those experiments to report one *P*s per published study, only including those experiments relevant to the question (e.g., mealybug life stage).

Earlier life stages of mealybugs have higher reported transmission efficiency than more mature life stages. *Pl. ficus* first and second instar nymphs have reported *P*s = 0.04–0.2 (Tsai et al., 2008;Mahfoudhi et al., 2009), and adults have *P*s = 0.009–0.02, about 10-fold lower than the nymphs. *Ph. aceris* first and second instars have *P*s = 0.05 and 0.02, respectively (Le Maguet et al., 2012). *Ps. longispinus* first instar nymphs transmit GLRaV-3 at *P*s = 0.08, and *Ps. calceolariae* first instar nymphs have *P*s = 0.02, while no transmission was found by third instars of either mealybug species (Petersen and Charles, 1997). Because nymphs settle and feed more quickly than adults (Sandanayaka et al., 2012), it is possible that transmission by adult mealybugs would increase with longer access periods, although most studies appeared to use sufficiently long periods that this should not have confounded the results.

There also appears to be variation in transmission efficiency of GLRaV-3 among mealybug species (**Figure 2**). Three different research groups found similar *P*s values for *Pl. ficus* nymphs, ranging from 0.04 to 0.2 (Douglas and Krüger, 2008;Tsai et al., 2008,^2010^,^2011^;Mahfoudhi et al., 2009). Estimated *P*s for *Ph. aceris* was 0.004–0.03, for *Ps. calceolariae* 0.02, and for *H. bohemicus* 0.002–0.003 (Petersen and Charles, 1997;Sforza et al., 2003;Zorloni et al., 2006;Le Maguet et al., 2012); but different life stages were used and the results are probably not directly comparable. Widely variable results were obtained within *Ps. longispinus*, with transmission ranging from *P*s = 0.08–0.38 (Petersen and Charles, 1997;Kuniyuki et al., 2005;Douglas and Krüger, 2008). The variation found within *Ps. longispinus* and among studies in general could be due to varied experimental techniques, to differences in transmission efficiency among insect populations or species, or to differences in GLRaV-3 variants that were tested.

It is not known whether GLRaV-3 populations within a donor plant affects transmission by mealybugs, but many viruses are transmitted at higher rates when the donor plant has higher viral infection (Froissart et al., 2010). GLRaV-3 populations vary seasonally in magnitude and distribution within a host plant, but the general trends are not well understood; virus population in leaves may increase during the growing season before dropping as leaves senesce (Tsai et al., 2012). Differences in transmission efficiency when mealybugs either acquire from, or inoculate to different plant tissues have not been found, although there is evidence that acquisition from stems may lead to lower transmission than from petioles or leaves (Tsai et al., 2011). Transmission by *Ps. longispinus* and *Ps. calceolariae* nymphs, for example, was tested early and late in the growing season from known infected vines in a vineyard, and no difference was found between the two time points (Petersen and Charles, 1997). While a change in transmission with viral population, plant tissue, or season has not been found, this possibility should not be ignored.

## DISEASE ECOLOGY

Evidence of GLD spread in vineyards was first found in South Africa (Engelbrecht and Kasdorf, 1985), and confirmed there using an interplant study with healthy vines among established infected vines (Engelbrecht and Kasdorf, 1990). A similar interplant study in Spain also provided evidence of GLD spread (Cabaleiro and Segura, 1997a;Cabaleiro et al., 2008) following observations that older vineyards tended to have higher GLD incidence. In both cases mealybugs were recorded present at the interplant study sites. Controlled greenhouse tests of GLRaV-3 transmission by *Pl. ficus* (Engelbrecht and Kasdorf, 1990) and *Pl. citri* (Cabaleiro and Segura, 1997b) linked mealybugs to the observed vineyard spread. GLD spread in established vineyards, 8–10 years after the initial planting, has been documented in Australia (Habili et al., 1995;Habili and Nutter, 1997), California-USA (Golino et al., 2008), and France (Le Maguet et al., 2013). The rate of spread was similar in these studies, close to 10% increase per year once GLD infections were identified as being present, and newly infected vines were spatially aggregated, indicating vine-to-vine spread.

**FIGURE 3:**
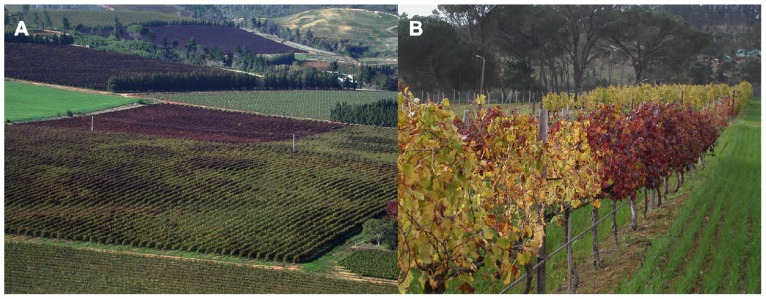
**(A)** Vineyards with high GLD incidence (dark red) serve as source of inoculum for adjacent blocks, in which disease spatial distribution is patchy, suggesting initial introduction of virus into uninfected blocks followed by within-block spread. **(B)** Example of secondary spread within rows, where an initial infection spread to neighboring plants. Both photographs were taken from the wine-producing region of Western Cape, South Africa.

Leafroll spread through newly planted blocks adjacent to highly infected blocks has been documented in South Africa (Pietersen, 2006; **Figure 3**), New Zealand (Charles et al., 2009), and Italy (Gribaudo et al., 2009). In New Zealand, populations of *Ps. longispinus* were monitored in nearby older leafroll-infected blocks, and the number of newly infected vines tended to increase more dramatically one growing season after large mealybug populations were found in neighboring blocks with 100% GLD incidence. Spatial analysis indicated that infected vines were randomly distributed throughout the blocks in early years, but aggregated toward the end of the study, indicating that long distance dispersal, such as wind-borne crawlers, as well as vine-to-vine movement of mealybugs was contributing to leafroll spread. In Italy, 20% virus prevalence was found 10 years after planting, indicating notably less apparent spread than in other regions (Engelbrecht and Kasdorf, 1990;Habili et al., 1995;Pietersen, 2006;Cabaleiro et al., 2008;Charles et al., 2009).

Grapevine leafroll disease is caused by a number of virus species, and within those species, there are genetically distinct variants. Within a growing region, for example, the geographical distribution differs among genetically distinct GLRaV-3 variants (Jooste et al., 2011;Sharma et al., 2011), yet little is known about what processes have led to this variation, or its impact on GLD. Furthermore, mixed variant infections within one plant are common and differential transmission of the variants may occur. In this complex system, interactions need to be considered among multiple virus and vector species. Potential virus and vector exchange with neighboring unmanaged communities needs to be evaluated. The effects of abiotic factors such as climate and nutrient availability need to be considered. Finally, a holistic view of the effects of various management practices is needed.

GLRaVs and their variants may vary in severity and may interact with each other during transmission and establishment in the host (Jooste et al., 2011). Some studies have also implicated GLRaV-1, -3, -4, and -9 in facilitating transmission of *Grapevine virus A* (GVA, *Vitivirus*;Zorloni et al., 2006;Hommay et al., 2008;Tsai et al., 2010;Le Maguet et al., 2012;Herrbach et al., 2013) but the evidence is inconclusive. These and other potential interactions could lead to changes in symptomatic disease prevalence and spread in vineyards. Some plant viruses can actually be beneficial to plants (Roossinck, 2011), and environmental conditions can alter the nature of effects a virus has on its host. The responses of GLD severity to varied environmental conditions, some of which can be controlled by changing management practices, remain largely unknown. Specific horticultural practices that are expected to affect the impact of GLD on yield and fruit quality should be studied. For example, partial defoliation of vines, which is expected to improve ripening, has been shown to improve the quality of must (freshly pressed fruit juice) from grapes infected with GLRaV-3 (Pereira-Crespo et al., 2012).

Pathogen-vector specificity can affect regional patterns of disease caused by vector-borne pathogens. Different genetic variants of a pathogen can differ in transmission efficiency by one vector species (Power, 1996;Tsetsarkin et al., 2011). Alternatively, one virus can be transmitted more or less efficiently by different vector species. GLRaV-3 is transmitted by many vector species and can be regarded as a “vector generalist” (Tsai et al., 2010), but GLRaV-3 transmission efficiency can differ among vector species (Douglas and Krüger, 2008). Adaptation to a vector that is already present, or introduction of a new vector into an area, can lead to dramatic changes in the prevalence of a vector-borne pathogen (Purcell and Feil, 2001). Furthermore, introduction of a new vector with a higher transmission efficiency of one pathogen variant than another can lead to changes in the relative prevalence of pathogen variants in a region, which can be as devastating as the introduction of a new pathogen. More knowledge is needed about the interactions of GLRaVs with their vectors.

## CURRENT DISEASE MANAGEMENT OPTIONS

Despite the economic impact of GLD on the world’s wine industry, efforts to manage this disease are still being developed or have only recently been implemented over large agricultural areas (e.g.,Pietersen et al., 2013). Here we provide a summary of current management strategies being utilized or tested in four countries – South Africa, New Zealand, California-USA, and France. Our goal is to highlight management options that have been used to address both shared and unique challenges associated with this disease, with the expectation that each case study provides novel insights into the complexities of controlling GLD in the field.

## A CASE STUDY OF DISEASE MANAGEMENT OPTIONS – SOUTH AFRICA

In South Africa, GLRaV-3 is the most important virus causing GLD (Pietersen and Kasdorf, 1993) and is transmitted predominantly by *Pl. ficus* and to a lesser extent by *Ps. longispinus* and multiple soft scale insect species (Walton and Pringle, 2004;Douglas and Krüger, 2008;Krüger and Douglas, 2013). Management of GLD is primarily through the provision of healthy planting material via the South African Vine Improvement Association (VIA). The VIA supplies the majority of planted vines utilized in the industry, and all VIA wine grape cultivars or clones are subjected to virus elimination via heat therapy and *in vitro* meristem tip propagation (Engelbrecht and Schwerdtfeger, 1979). Hardened off plantlets are established and maintained in insect-free greenhouses as *nuclear plants* (i.e., plant material of the highest level of sanitation in the certification scheme). On establishment, and every 5 years thereafter, these plants are subjected to compulsory tests for GLRaV-1, -2, and -3 (Goszczynski et al., 1995), *Grapevine fanleaf virus*, GVA and GVB by ELISA and by immunoelectron microscopy (Pietersen and Kasdorf, 1993) for GLRaV-4 and -5 in addition to the previously listed viruses. Furthermore, these plants are subjected to hardwood indexing on seven *Vitis* indicators (for 2 or 3 years depending on the disease). For plants to be certified as nuclear material, they must be negative for all viruses tested as well as GLD, grapevine stem grooving disease, grapevine corky bark disease, Shiraz disease, grapevine fleck disease, grapevine vein necrosis, and grapevine vein mosaic disease.

Planting material from nuclear blocks is propagated to establish *foundation blocks*, either in greenhouses or open field plantings. Open field foundation block vineyards must be on virgin soil (i.e., not previously planted in grapevines) that must test free of *Xiphinema index* (California dagger nematode), and must be at least 25 m from other vineyards. The vines from these blocks are tested every year by ELISA for GLRaV-1, -2, and -3 if they are located in high risk areas (less than 25 m from other grapevines if mealybugs are recorded in the vicinity) or every 3 years if in low risk areas (no mealybugs trapped or observed, and the block is at least 25 m from vineyards of lower phytosanitary status). Plants testing negative for GLD in foundation blocks may be used to establish *mother-blocks*. Mother-blocks are typically commercial grape-growing vineyards and only need to be 3 m away from other vineyards. They can be planted in untested virgin soil or on soil that has previously been planted to *Vitis* but tests free of *X. index*. Visual inspection for GLD symptoms is conducted annually in autumn on red cultivars, Chardonnay, Cape Riesling, and Semillon.

The use of certified planting material and the above plan, however, do not rid South African vineyards of GLD. Mother-blocks in traditional grape production areas become infected rapidly with GLD. For example, during a 2001–2006 spatio-temporal study of 55 red cultivar mother-block vineyards in which no specific GLD control was applied, once GLD infections were initially found there was an average annual GLD increase of 1.94 times (Pietersen, 2006). Because of this, South African mother-blocks are only utilized for planting material if GLD infection levels of less than 5% exist in the vineyard. At infection levels below 5%, the producer may permit the removal of infected vines, or canes from infected and single adjacent vines within the row may be cut and dropped annually before planting material is collected. In spite of these measures, GLD-infected planting material can still be found within the certified material, with randomly occurring GLD-infected vines in newly established vineyards observed in 3% of all the mother-blocks (Pietersen, 2006). Based on the average rate of infection amongst the 55 mother-blocks monitored, it was estimated that the initial GLD incidence in the planting material was less than 1%. Since the mid-2000s many local plant improvement organizations have been propagating mother-block material in areas in which grapevines have not been grown previously. Certified material is therefore now differentiated as mother-blocks in low risk areas (three-star rated material) and in areas at risk to GLD re-infection (one-star material). At this time, three-star material is still relatively scarce; therefore responsible producers apply systemic insecticides at planting, and rogue GLD-infected vines in the newly established vineyards.

Secondary spread from a GLD-infected vine to adjacent vines in a row is the major cause of new GLD infections in the industry and occurred in all mother-blocks monitored (Pietersen, 2006; **Figure 3**). Roguing of infected vines is feasible and effective on an experimental scale (Pietersen et al., 2003). Removal of infected vines, combined with mealybug control, is extremely effective at controlling GLD in commercial vineyards, and this practice is becoming more widely applied (Pietersen and Walsh, 2012).Pietersen (2006) also presented circumstantial evidence of GLD spread in a replanted vineyard from a preceding vineyard, either through the presence of viruliferous mealybugs on remnant root material, or on volunteer hosts. The persistence of GLRaV-3 in remnant roots and potential of transmission by mealybugs from these has subsequently been demonstrated (Bell et al., 2009). Fallow periods of up to two seasons, during which remnant roots are removed, have been utilized in a number of commercial vineyards locally (Pietersen and Walsh, 2012). A clear demonstration of the efficacy of this strategy on its own must still be shown.

Gradients of GLD infection from the edges of a vineyard are commonly observed.Pietersen (2006) recorded gradients of various slopes from 70% of the 55 mother-blocks analyzed. These gradients reflect initial introduction of the virus from a source external to the vineyard, and in 32% of the blocks monitored the gradient could clearly be ascribed to an adjacent GLD-infected vineyard. These gradients are likely due to immigrating first instar mealybugs, either by their own motility over short distances, or on farm workers’ clothing, on implements, by wind, ants, or possibly even by birds. A number of strategies have been employed to reduce the introduction of the disease from external sources (Pietersen and Walsh, 2012), including stringent control of mealybugs in all vineyards within the region, planting new vineyards far from heavily infected vineyards, avoiding traffic (implements and workers) from infected to healthy vineyards, or if unavoidable, washing implements with soapy water when moving between vineyards, and conducting work in healthy vineyards before moving into an infected vineyard. Following such a program, the near-eradication of GLD has been achieved at a commercial wine estate in the Somerset West district, from 100% infection on 41.26 ha in 2002 to 0.027% infection on 77.84 ha in 2012 (Pietersen et al., 2013). This result provided strong evidence that by using the full suite of GLD and mealybug control strategies available, disease incidence and its progression can be reversed. Further studies are required to determine the relative efficiency of individual components of the integrated control strategy.

## A CASE STUDY OF DISEASE MANAGEMENT OPTIONS – NEW ZEALAND

Grapevine leafroll disease was first described in New Zealand in the early twentieth century (Bragato, 1902), but it was not until the 1960s that research to quantify its impact on vine performance and fruit quality started (Chamberlain et al., 1970;Over de Linden and Chamberlain, 1970). Today, GLRaV-3 is the most widespread and economically damaging disease affecting grapevines (Bonfiglioli and Hoskins, 2006). Concerned with the long-term impact of GLRaV-3 on wine quality, the national sector body, New Zealand Winegrowers (NZW), developed the grafted grapevine standard, with one of its aims being to minimize the probability of plant material with diseases such as GLRaV-3 being released to the industry.

A grower survey in 2005 revealed few respondents were well informed about the threats posed by GLRaV-3 or the options available for limiting its spread. Furthermore, a review of local and international literature, aimed to identify GLRaV-3 research priorities and knowledge gaps, was prepared (Charles et al., 2006). Of the numerous recommendations generated by these NZW initiatives, a plan for grower education and communication was prioritized. A collaborative program was established in which viticulturists, winemakers, and vine nursery groups collaborated with plant virologists, vine physiologists, and entomologists in a multi-disciplinary integrated approach to establish a GLRaV-3 control program.

A GLRaV-3 control pilot project began in 2009 in two North Island winegrowing regions: the Gimblett Gravels, a winegrowing sub-region in Hawke’s Bay, and Martinborough. The project focused on controlling GLRaV-3 in red grape varieties because symptomatic vines are relatively easily identified visually by the dark red downward curling leaves with green veins. The project had three aims: (1) to visually identify and map the presence of GLRaV-3 in vines in both regions; (2) to control GLRaV-3 through a combination of vine removal, hygiene practices, and improved vector management; and (3) to enable eventual vine replacement, whilst incorporating the new knowledge into “best practice” guidelines for nationwide dissemination (Hoskins et al., 2011). Here, we summarize the process and some of the achievements of the first 3 years of a 6-year project.

The control of GLRaV-3 in the field has focused on two strategies. The first was the removal (or roguing) of individual symptomatic vines (or small clusters of symptomatic vines), with most vineyard owners roguing symptomatic vines only. The second strategy, whole block removal, was adopted in blocks where roguing individual vines was considered unlikely to contain or control the disease. In New Zealand, the economic threshold of GLD incidence beyond which roguing was thought to be practical was ~20% of vines (Hoskins et al., 2011).

In the Gimblett Gravels and Martinborough regions, participating vineyards supplying ~40 individual wineries encompassed an area of ~1,100 ha. Training of vineyard personnel to identify GLRaV-3 symptomatic vines accurately was initiated. Once trained, vineyard personnel systematically moved through every red grape variety block late in the season identifying symptomatic vines, plotting their position with GPS and marking vines to guide the roguing done in winter. While the regional mapping of symptomatic vines is ongoing and the data have yet to be fully interpreted, individual vineyard owners are provided annually with preliminary block-specific results. The provision of this information has substantially aided the profile of the project and its educational goals, particularly in measuring the incidence and changes to the spread of GLRaV-3 (Hoskins et al., 2011).

Augmenting the regional mapping of GLRaV-3 were block-specific studies focused on GLRaV-3 identification together with monitoring the disease vectors, mealybugs. Data were analyzed from nine blocks in the Gimblett Gravels planted in various red grape varieties (~21,000 vines). The objective was to determine if a combined approach of GLRaV-3 visual identification and roguing, supported by good vector control could reduce disease incidence to a point where less than 1.0% of vines per block were rogued annually. While this study is still underway, preliminary results are presented here (V. A. Bell, unpublished results).

In the nine study blocks, the percentage of symptomatic vines identified and rogued per year steadily declined from an average of 11.8% in 2009 to 2.7% in 2012. Over this period, a total of 4,902 symptomatic vines were rogued across the nine study blocks (23.8% of the original plantings). After 3 years, the evidence suggests roguing can successfully control GLRaV-3, although as discussed, good vector management was integral to a successful outcome.

In 2011 and 2012, mapping the positions of symptomatic vines in each block revealed 82.4 and 88.6%, respectively, were in close proximity to a vine rogued since 2009, supporting similar findings in earlier studies (Habili and Nutter, 1997;Cabaleiro and Segura, 2006;Pietersen, 2006). Of these neighboring vines, most at risk of acquiring GLRaV-3 were the “first” vines, the within-row immediate neighbors of a vine rogued at least 12 months earlier. This pattern of GLRaV-3 spread suggested the infection pathway, mediated by vector dispersal, was from the vine rogued at least 12 months earlier. In 2010, an average of 78% of all “first” vines had no visual symptoms of GLRaV-3, indicating they were either healthy or if infected, the visual symptoms were yet to express. By 2012, “first” vines relative to other “nearest neighbors” remained most at risk of GLRaV-3, although on average, 92% of “first” vines were symptomless. Based on the results of this study, the risk of a “first” vine acquiring GLRaV-3 was low, especially as the benefits of roguing and effective vector management accumulated over time. Consequently, good control of GLRaV-3 was achieved under almost all circumstances by roguing symptomatic vines only.

A further important aspect of the project was to determine the extent to which vector populations influenced GLRaV-3 control outcomes. Throughout this study, the vector most commonly encountered was the mealybug *Ps. calceolariae*, which colonizes all aerial parts and the roots of grapevines. Monitoring indicated mealybug numbers declined in most blocks over time as vineyard managers heeded warnings to improve vector control and to adopt better hygiene practices, such as removing the remnant roots of rogued vines. Being long-term reservoirs of GLRaV-3 (Bell et al., 2009), remnant roots colonized by *Ps. calceolariae* provide a likely pathway for the disease to infect young replacement vines.

In 2012, GLRaV-3 incidence in three of the nine study blocks was reduced to less than 1.0%, and in these blocks since 2010, mealybug counts from the third and final generation in late summer (March) were consistently low, ranging from two to eight mealybugs per 100 vine leaves inspected. Significantly, in two of these blocks (identified as A and B), disease incidence in 2009 was relatively high at 10.1 and 16.0%, respectively, so to have effectively controlled GLRaV-3 in just 3 years was an encouraging result. Given the known economic impacts of GLRaV-3, it was not possible to include an “unmanaged control” component in any of the study blocks. Despite this position, the finding of significant mealybug populations (78–175 mealybugs per 100 vine leaves inspected) in another two study blocks (C and D) provided useful comparisons with blocks A and B. In 2009, GLRaV-3 incidence in blocks C and D was 9.9 and 15.1%, respectively, but by 2012 cumulative vine losses due to GLRaV-3 were ca. 40%, culminating in the removal of all residual vines in both blocks.

With symptomatic vines identified and rogued each year in all nine study blocks, what most distinguished blocks C and D from the other seven was the high number of mealybugs. In this instance, poor mealybug control was probably due to non-adherence to insecticide (i.e., buprofezin) best practice with water volumes about one-third the label recommendations, thus compromising coverage and vine wetting. These contrasts in vector abundance demonstrated that roguing symptomatic vines alone provided relatively unsuccessful control of GLRaV-3 when it was not supported by effective mealybug management. In other words, while total eradication of *Ps. calceolariae* was not a prerequisite for controlling GLRaV-3, containing this disease was only achieved in those blocks where mealybug numbers were consistently low.

## A CASE STUDY OF DISEASE MANAGEMENT OPTIONS – CALIFORNIA-USA

California accounts for 89.5% of domestic U.S. wine grape production – a total of 3.6 million tons in 2010 – with a farm gate value of US$2.06 billion. In a survey conducted by the American Vineyard Foundation in 2009, grape growers considered mealybug control and GLD one of their top priorities, solidifying this as a high priority research issue that threatens the sustainability of the industry. California grape growers have begun implementing multiple tactics in an effort to minimize current and future losses attributed to GLD. Although various GLRaV species are present in California, GLRaV-3 has been identified as the most important in the premiere wine-producing region of Napa Valley (Sharma et al., 2011).

California growers aim to minimize incidence of GLD and other grapevine diseases by planting material certified through the California Grapevine Certification and Registration (CGC&R) Program. Established in 1956, the CGC&R Program is administered by the California Department of Food and Agriculture (CDFA;Alley and Golino, 2000). It targets the elimination of grapevine diseases that spread from vine-to-vine by grafting and/or vegetative propagation. Under the auspices of the CGC&R Program, correctly named grape materials that pass specific disease tests are identified and/or created, and maintained as Foundation materials by Foundation Plant Services (FPS) at the University of California, Davis, for use by California commercial nurseries.

The CGC&R Program includes provisions for three levels of planting stock: California Foundation stock, California Registered stock, and California Certified grapevines. FPS at University of California, Davis maintains vines in the FPS Foundation block; materials derived from FPS Foundation vines are California Foundation stock. Vineyards planted by participants in the CGC&R Program using California Foundation stock material are known as California Registered increase blocks. They are inspected annually and tested for pathogens as needed by inspectors from CDFA. Material derived from the California Registered increase blocks is California Registered stock. When California Registered cuttings are rooted, or California Registered scion cuttings are grafted to California Registered rootstock cuttings, the resulting vines are classified as California Certified grapevines and are sold to growers for commercial planting. Nursery participation in the CGC&R Program is strongly encouraged but not mandatory. Other limitations to the CGC&R Program include the use of traditional screening methods (ELISA, RT-PCR, qPCR), which require prior knowledge of pathogens and are incapable of detecting unknown variants or agents. The variable population of GLRaV species in plant tissue, including rootstock and scion, is also a limitation to the production of reliable laboratory test results, and therefore material that is free of known viruses.

To manage GLD spread, California wine grape growers identify symptomatic vines, document annual changes in disease incidence in vineyard blocks, and remove diseased vines. Vine removal occurs only in blocks where disease incidence is below a threshold determined by each grower. Thresholds are typically generated by an economic analysis based on vineyard age, cost of replanting versus redevelopment, grape purchasing contracts, the wine program for which the grapes are destined, and other considerations. Generally, growers identify vineyards with greater than 20–30% disease incidence for redevelopment of the entire block, whereas vine removal occurs in vineyards with less than 20% disease incidence. However, the threshold for roguing versus redevelopment varies considerably among growers, especially when grapes are destined for a high price point wine, or when redevelopment is particularly costly or challenging, such as in hillside blocks. Timely removal of diseased vines is limited by the cost associated with routine and reliable identification of these vines. It is not common practice in California to regularly identify and rogue symptomatic vines, although some growers have made it a regular practice in recent years. Dedicating resources to this effort can be complicated because peak symptom development overlaps with harvest period. There has also been a general lack of awareness of the importance of this practice. Both concerns are being addressed through research and educational programs directed by researchers at the University of California, with the goal of increasing awareness of the importance of this practice while identifying faster and easier ways to do it. In particular, infected vines may be identified using hyperspectral imaging technology that measures differences in leaf spectral reflectance between GLRaV-3 infected and uninfected grapevines (Naidu et al., 2009).

Mealybug management is a major component of GLD control programs in California. Currently five mealybug species cause direct damage and are potential vectors of GLRaV in vineyards: *Pl. ficus*, *Ps. maritimus*, *Ps. viburni*, *Ps. longispinus*, and *Ferrisia gilli* Gullan (Daane et al., 2012). Recently, a multiplex PCR procedure was developed to identify seven species of mealybug typically found in California vineyards (Daane et al., 2011). The ability to identify young mealybug nymphs to species using rapid and sensitive detection techniques helps growers make informed decisions about mealybug management. Trapping programs using pheromone-loaded lures also provide important information on mealybug species presence.

Growers rely on a combination of tactics including insecticides, mating disruption, biological control, and management of some ant species to minimize populations of *Pl. ficus* (Daane et al., 2008). Unfortunately, the Argentine ant (*Linepithema humile*) in particular, which “farms” mealybugs, is very aggressive in California vineyards and growers therefore struggle to maintain the extremely low mealybug populations required to minimize virus transmission. Results of recent investigations suggest that regional management programs for *Pl. ficus*, utilizing a combination of these tools, may provide better long-term control than individual efforts by isolated growers. Efforts are therefore underway to develop and implement similar regional management programs for other vineyard mealybug species. Populations of *Ps. maritimus* are of particular interest in coastal northern California vineyards, where they are commonly associated with spread of GLRaV-3, the most prevalent virus species that is spreading in the area.

## A CASE STUDY OF DISEASE MANAGEMENT OPTIONS – FRANCE

In France, GLD is believed to be present since at least the early 1900s, as “rougeau” or “rougeot,” and later as “enroulement foliaire,” suspected then to be the same as the “*Rollkrankheit*” and “Leafroll” already described in Germany and the United States, respectively (Goheen et al., 1958;Vuittenez, 1958). However, GLD has long been seen as an unimportant problem for French viticulture, at least less crucial than fungal diseases and even *Grapevine fanleaf virus*. One reason may be that GLD symptoms were, and still are, often confused with other diseases or deficiencies, especially on white-berried cultivars. However, management of GLD was soon seen as a matter of sanitary selection (Vuittenez, 1958). At present, three species, GLRaV-1, -2, and -3, are detected in French commercial vineyards. GLRaV-1 and -3 are more frequent in north-eastern (Alsace, Beaujolais, Bourgogne, Champagne) and in southern (Mediterranean regions and Bordelais) vineyards, respectively, whereas GLRaV-2 is more common in the south-west. Over the last decades, infections by GLRaV-1 were recorded from many areas in Burgundy, Beaujolais, and Champagne. At the same time, wider infestations of mealybugs and soft scales were reported from these regions, probably related to the decreasing use of insecticides against the European grapevine moth (*Lobesia botrana*).

In France, sanitary selection was set up in the 1940s with the aim of producing healthy plant material to initially combat the spread of *Grapevine fanleaf virus* (Valat, 1972;Walter and Martelli, 1997). This process was greatly improved since then, due to progress in virological knowledge and detection methods, and is still seen today as the primary way to control GLD, which was incorporated into the system at a later date, among other viral and phytoplasmal diseases. According to French regulations (see www.legifrance.com), which follow a European Commission Directive, all planting material is classified in one of four categories: *initial*, *base*, *certified*, or *standard* (FranceAgriMer, 2013). The first three are produced only by specific institutions (initial, base) or nurseries (certified) and are subjected to detection tests to demonstrate the absence of viruses. Indexing is performed for any new clone prior to registration. During the pre-multiplication and multiplication processes, ELISA tests are used for all certified material. So far, only GLRaV-1 and -3 are taken into account among the GLRaVs, and all vines found infected at these steps have to be removed. The production of initial, base, and certified material is under the control of FranceAgriMer, a government agency. Growers can choose between certified and standard planting material, the first being more expensive but tested free of certain viruses (Walter and Martelli, 1997). Standard material is produced either in nurseries, where only visual inspections of symptoms are performed, or by growers who practice mass selection. Therefore, the use of standard material increases the risk of spreading GLD.

The use of insecticides against GLRaV vectors is allowed in France. However, few active ingredients, mainly organophosphates, are specifically registered for controlling scale and mealybug insects on grapevine. Moreover, insecticide implementation is not regulated nationally and will vary according to regional practices and viticultural advisers. Deeper biological and epidemiological knowledge is urgently required in order to adjust the use of insecticides to specific disease risk levels, depending on disease incidence and vector density. While natural enemies of vectors are known and experiments (e.g., using lacewings) are underway, there is no biological control program established in France. The development of vector monitoring by lure traps and mating disruption will first require the identification of the sex pheromones, still unknown, of common species like *Ph. aceris*, *H. bohemicus*, and *Pa. corni*.

In the course of certification schemes, thermotherapy- or meristem culture-based sanitation methods are sometimes used, especially for high value clones or cultivars. GLD management in France currently relies mainly on the sanitary selection of planting material, so in the long-term, healthy planting material seems the key to controlling GLD. More effort is to be devoted to improve sanitary selection, requiring deeper knowledge of the diversity of viruses and their effect on grapevine. Virological knowledge will also improve both specificity and sensitivity of detection methods used. Moreover, it should be desirable in the future to coordinate the certification schemes among countries producing planting material. In France, growers need to be better informed about GLD symptomatology and the detrimental effects of GLD, especially in case of co-infection of vines with two or more distinct viruses, a common phenomenon for grapevine. In addition, better information should assist nurseries and others producing plant material, particularly those using mass selection, to adopt procedures aimed at producing virus-clean grapevines. Moreover, a recent French study showed the risk to neighboring vineyards posed by leafroll-infected and scale insect-infested plots (Le Maguet et al., 2013). Therefore, a new plantation should take into account the sanitary status of neighbors and the possible spread of vectors from older to younger plots. Better guidelines (such as planting of hedges, vine-free strips between plots, insecticide treatments) should be tested for their efficacy, particularly for isolating mother plant blocks.

Finally, the wide variety of GLD epidemiological scenarios in France (e.g.,Le Maguet et al., 2013) and the difficulty to define any damage thresholds hamper determining recommendations on how best to manage virus-infected and/or vector-infested vineyards. There is a crucial need for deeper knowledge of epidemiology, including determinant factors such as infection intensity, sensitivity of cultivars, virus and vector species present, and landscape structure. GLD management is more than a single grower’s concern, instead requiring a collective approach across whole communities of growers, advisors, and scientists.

## MANAGEMENT STRATEGIES – SUMMARY

The preceding case studies from grape-growing regions worldwide share remarkable similarities, and illustrate a combination of approaches required for GLD control. Importantly, they illustrate that GLD must be managed at a large scale and that a long-term management strategy is needed. Especially in the case of GLRaV-3, infected vines or blocks will continually act as sources of inoculum, perpetuating disease spread. For this reason, a coordinated area-wide approach is required, with education of growers as the first step. A second component of control is access to uninfected propagation material. A centralized service that includes a stringent certification program is needed to provide disease-free planting material for a growing region, as is the case in the countries reported here. In some regions where vectors may live on infected roots from previous crops, extra care is needed to assure that the planting area does not contain a GLRaV-3 source in the remaining live roots of infected vines that were removed. In both newly planted blocks and those with mature vines, roguing of symptomatic vines, and possibly vines immediately adjacent to those symptomatic vines, appears to be effective in preventing future disease spread. A third aspect of GLD management is control of insect vectors. Because mealybug nymphs are the most infective life stage and could travel long distances in air currents, insect control is often needed before large mealybug populations are detected. Therefore, knowledge of the life cycle of vectors can inform decisions regarding implementation of insect control. Finally, effective control of an existing GLD problem cannot be achieved within one growing season. Instead, favorable results are found after multiple years of regional management practices that incorporate eliminating infected plant material and controlling vector populations. Still lacking in GLD management is fundamental knowledge about disease spread. While a majority of management has focused on red varieties, largely because identification of symptoms is easier, GLD also affects white varieties. As long as white varieties continue to be overlooked, they may continue to be a source of virus thus hampering management efforts.

## Conflict of Interest Statement

The authors declare that the research was conducted in the absence of any commercial or financial relationships that could be construed as a potential conflict of interest.
